# Enhancing Meniscal Repair: Investigating the Impact of an Exogenous Fibrin Clot

**DOI:** 10.7759/cureus.53083

**Published:** 2024-01-27

**Authors:** Chrysanthos Chrysanthou, Nikolaos Laliotis, George K Paraskevas, Nikolaos Anastasopoulos, Gregory Packer

**Affiliations:** 1 Orthopaedics and Traumatology, Interbalkan Medical Center, Thessaloniki, GRC; 2 Anatomy and Surgical Anatomy, Aristotle University of Thessaloniki, Thessaloniki, GRC; 3 Orthopaedics, Interbalkan Medical Center, Thessaloniki, GRC; 4 Orthopaedics, Aristotle University of Thessaloniki, Thessaloniki, GRC; 5 Orthopaedics and Trauma, Mid and South Essex NHS Foundation Trust, Southend-on-Sea, GBR; 6 Orthopaedics, Spire Wellesley Hospital, Southend-on-Sea, GBR

**Keywords:** meniscal repair with exogenous fibrin clot augmentation, exogenous fibrin clot preparation, meniscal repair techniques, fibrin clot augmentation, fibrin clot

## Abstract

This study conducted a comparative analysis of meniscal rupture repair, evaluating outcomes with and without the application of an exogenous fibrin clot to enhance meniscus repair. The research incorporated a relatively large sample size (24 patients) and employed a randomized control group with similar age characteristics and morphological types of meniscal ruptures as the study group. Notably, two postoperative follow-up times, at the third and 12th postoperative months, were utilized, distinguishing this study from related research.

In the third postoperative month assessment, the fibrin clot technique demonstrated a significant advantage over simple stapling, as evidenced by markedly improved Tegner Lysholm Knee Scoring Scale (TLKSS) and Modified Cincinnati Rating System Questionnaire (MCRSQ) clinical assessment scores. Subsequent MRI scans at 12 months post-treatment revealed a high rate (91.67%) of complete healing in menisci treated with a fibrin clot, with only 4.17% exhibiting incomplete healing. This study expanded on previous research by including longitudinal ruptures and bucket-handle ruptures in addition to radial ruptures.

The findings highlight a notable early improvement (third postoperative month) in the clinical assessment of longitudinal and bucket-handle ruptures treated with a fibrin clot during meniscus repair. This research contributes valuable insights into the efficacy of fibrin clots in enhancing meniscus repair, suggesting positive clinical and radiological outcomes, especially in the early stages postoperatively.

## Introduction

In recent years, there has been a heightened appreciation for the pivotal role played by the menisci in knee function and biomechanics. Orthopedic surgeons frequently encounter meniscal pathology, a primary motivator for arthroscopic interventions. The recognition of the deleterious impact of meniscectomy on the chondral surface of the condyles has underscored the importance of meniscal repair in preserving knee joint health [1-3]. Consequently, the orthopedic community has explored various surgical techniques to aid and optimize the healing process after meniscal repair [4].

Among these techniques, the application of a fibrin clot derived from exogenous blood has emerged as a straightforward and cost-effective modality to enhance meniscal repair [5,6]. The formation of a fibrin clot, a relatively simple procedure, aligns with the broader objective of promoting efficient and robust healing in meniscal injuries. The use of fibrin clots in meniscus repair has garnered attention, with encouraging results that demonstrate statistically significant improvements when compared to conventional methods such as simple meniscus stapling [7-12]. This highlights the potential benefits of incorporating fibrin clots into meniscal repair procedures.

## Materials and methods

Inclusion criteria

To ensure sample homogeneity and minimize selection errors, strict inclusion criteria were applied (Table 1).

**Table 1:** Inclusion criteria.

Inclusion criteria in the study	
1	The age of the patient, the upper limit of which was set to 40 years
2	The absence of other ligamentous injuries that influence knee stability
3	A meniscal lesion concerns only one of the meniscus (medial or lateral)
4	The presence of osteoarthritis, rheumatoid arthritis, or other pathologies (bone bruising, osteonecrosis, tumors, etc.) constitutes an absolute contraindication
5	A contraindication is also the re-injury of the operated knee during the postoperative follow-up program

Patient selection

Initially, 63 patients were screened based on inclusion and exclusion criteria. From this cohort, 12 patients were subsequently excluded from participation.

Three individuals exhibited recurrent knee injuries resulting from sports activities. Moreover, these patients were already two months postoperative, making them ineligible for participation in the postoperative rehabilitation programme. Nine patients were lost to regular follow-up, introducing a potential impact on the continuity and reliability of the study. Following the exclusion process, the final study sample comprised 51 patients, who were randomly assigned to either Group A (control, n=27) or Group B (study, n=24). The meticulous screening and selection process aimed to ensure a homogeneous and reliable study population.

Demographics

Group A (control) comprised 23 males and four females, with an age range of 17-40 years (mean age 27.6±7 years), while Group B (study) consisted of 21 males and three females, with an age range of 18-40 years (mean age 28.9±6 years). The combined total was 44 males (86.27%) and seven females (13.73%), with a mean age of 28.2 years.

Lesion characteristics

Group A (Control Group): Fibrin Clot Was Not Used

Among the 27 cases of meniscal ruptures in Group A, 22 of them involved the medial meniscus, while the remaining five affected the lateral meniscus. Furthermore, in 20 cases, the meniscal lesions were located in the medial third, and in the remaining seven cases, they were situated in the posterior third of the meniscus. Twenty-two meniscal lesions were identified in the "red-white" zone, while five cases were found in the "white-white" zone. Regarding the morphology of the ruptures, nine were radial, nine were bucket-handle, six were longitudinal, and three were horizontal. The meniscal repairs involved the use of between one and five stitches, with an average of 2.4±1 stitches per case.

Group B (Study Group): Fibrin Clot Was Used

Among these, 20 cases involved the medial meniscus, while four involved the lateral meniscus. In 16 cases, the meniscal rupture occurred in the medial third, and in eight cases, it occurred in the posterior third of the meniscus. Additionally, 18 cases had meniscal rupture in the "red-white" zone, and the remaining six cases had rupture in the "white-white" zone. The classification of ruptures in this group included eight radial, eight bucket-handle, five longitudinal, and three horizontal cases. The meniscal repairs involved the use of between one and five stitches, with an average of 2.3±1 stitches per case.

In the combined cohort of 51 patients from both groups, 44 were males (86.27%) and seven were females (13.73%), with an average age of 28.2 years, and the predominant age range for the majority of patients was between 30 and 49 years.

Injury causes

Among the study participants, 29 patients experienced meniscal ruptures due to sports activities, 12 patients sustained injuries from falls resulting from abnormal support on uneven ground, five patients were involved in road traffic accidents (RTAs), three patients experienced work-related accidents, and two patients had injuries from falling from a height (Table 2).

**Table 2:** Causes of meniscal rupture. RTA: road traffic accident.

Causes	Number of meniscal ruptures
Sports activity	29
Fell on uneven ground	12
RTA	5
Work accident	3
Fell from height	2

Clinical and radiological examination

Each patient underwent a comprehensive clinical and radiological examination before meniscal rupture treatment (Table 3). After the surgical treatment, postoperative examinations were conducted at both the three-month and 12-month marks. These evaluations involved a thorough reassessment of clinical tests and questionnaires (Tegner Lysholm Knee Scoring Scale (TLKSS), Knee Society Scoring System (KSSS), Modified Cincinnati Rating System Questionnaire (MCRSQ)). Additionally, a magnetic resonance imaging radiological examination was performed, specifically at the 12-month follow-up.

**Table 3:** Preoperative screening. MRI: magnetic resonance imaging; TLKSS: Tegner Lysholm Knee Scoring Scale; KSSS: Knee Society Scoring System; MCRSQ: Modified Cincinnati Rating System Questionnaire.

Preoperative screening	
1	X-ray. It was performed to rule out any traumatic lesions or other knee pathologies
2	MRI of the knee to highlight the meniscal rupture as well as if any other ligament lesion is present
3	Apley and McMurray clinical tests
4	The evaluation is based on the following clinical questionnaires: (a) TLKSS; (b) KSSS; (c) MCRSQ

Statistical analysis

The statistical analysis encompassed the calculation of mean values and standard deviations for all parameters. To scrutinize the preoperative and postoperative results within each group, the paired-sample t-test was employed. Meanwhile, a comparison between the two groups was executed using the uncorrelated control t-test (independent sample t-test). The significance level was predetermined at p<0.05. For statistical processing, IBM SPSS Statistics for Windows, Version 17.0 (Released 2008; IBM Corp; Armonk, New York, United States) was utilized.

Surgical technique

**Figure 1:**
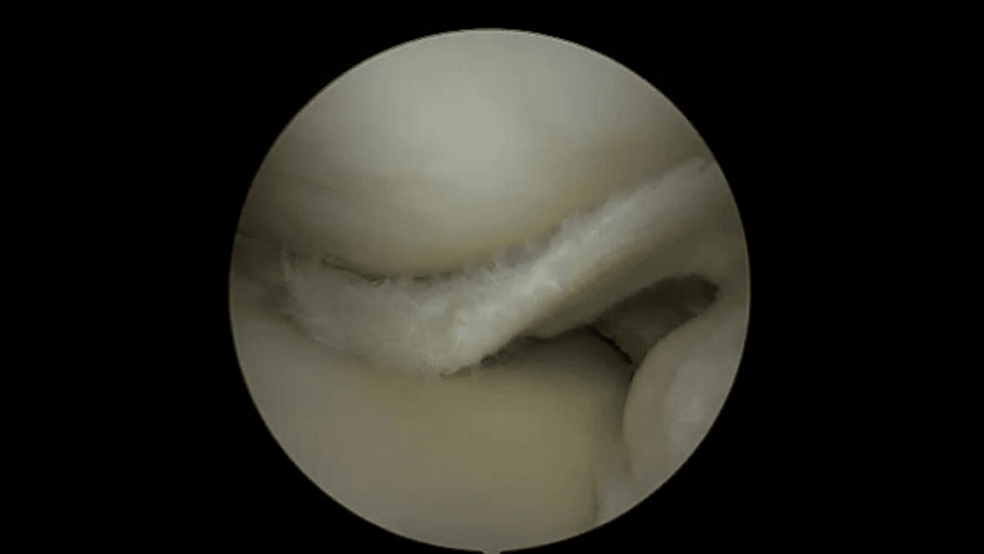
Illustration of a medial displaced meniscus with bucket handle rupture, depicting the detachment and flipping of meniscal tissue within the knee joint.

**Figure 2:**
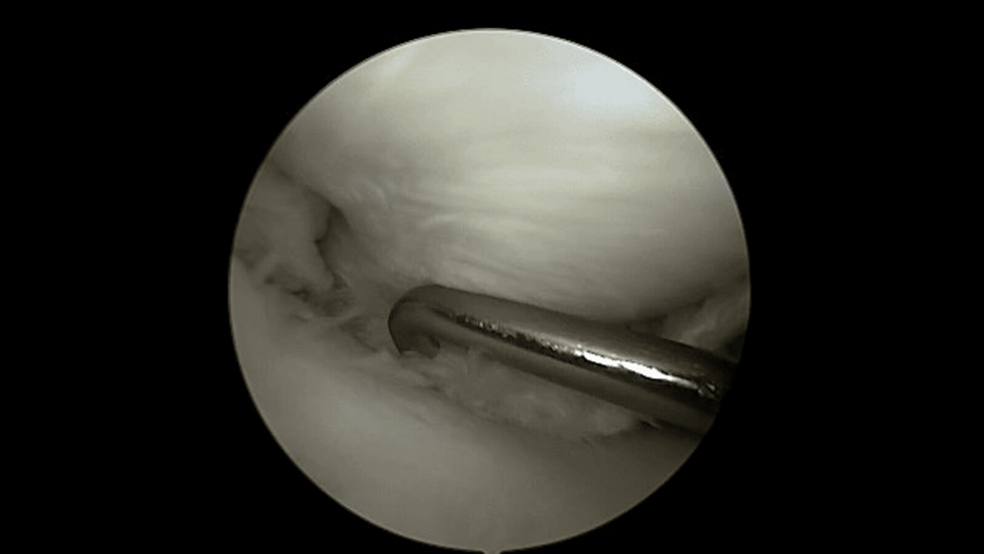
Reduction of the displaced tear using a surgical probe.

All surgical procedures were conducted by a consistent surgeon using either the inside-out or the all-inside technique for meniscal rupture repair. The patient was placed in a supine position on the operating table, and a diagnostic arthroscopy was performed to identify and classify the type of meniscal rupture. In cases of bucket-handle tears or displaced meniscal flaps, anatomical reduction of the tear was achieved using a surgical probe (Figures 1, 2).

**Figure 3:**
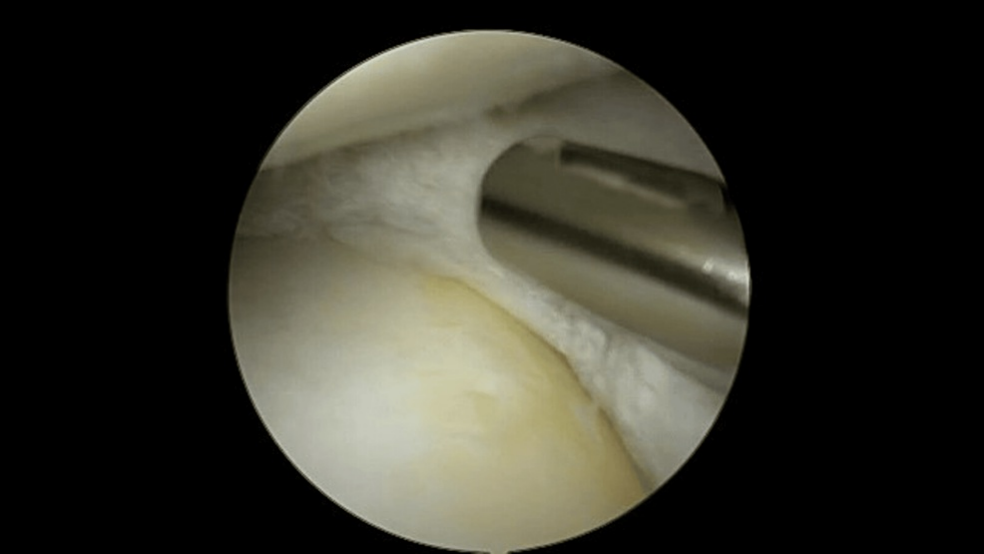
Debridement of the peripheral portion of the medial meniscus.

**Figure 4:**
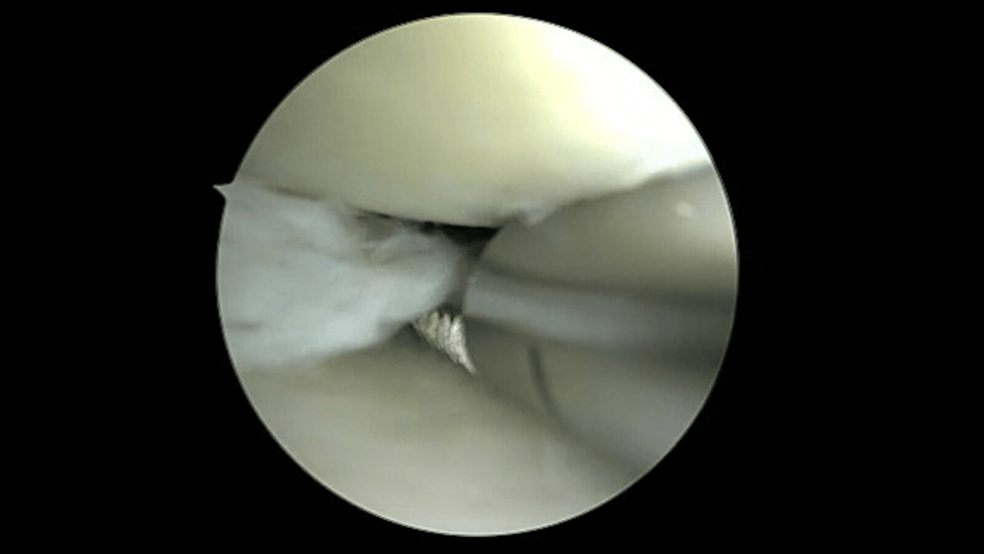
Debridement of the central portion of the medial meniscus.

Subsequently, the central and peripheral portions of the meniscal lesion were debrided using a rasp or a motorized shaver without suction to avoid additional damage to the meniscus (Figures 3, 4). Synovial abrasion was performed to enhance meniscal repair in a similar manner. At this point, the length of the meniscal gap was measured, and the number of sutures required for repair was determined, ensuring a spacing of approximately 1-1.5 cm between them. For patients in the study group (Group B), the length of the meniscal gap played a crucial role in shaping the fibrin clot according to the specific lesion.

Fibrin clot preparation

**Figure 5:**
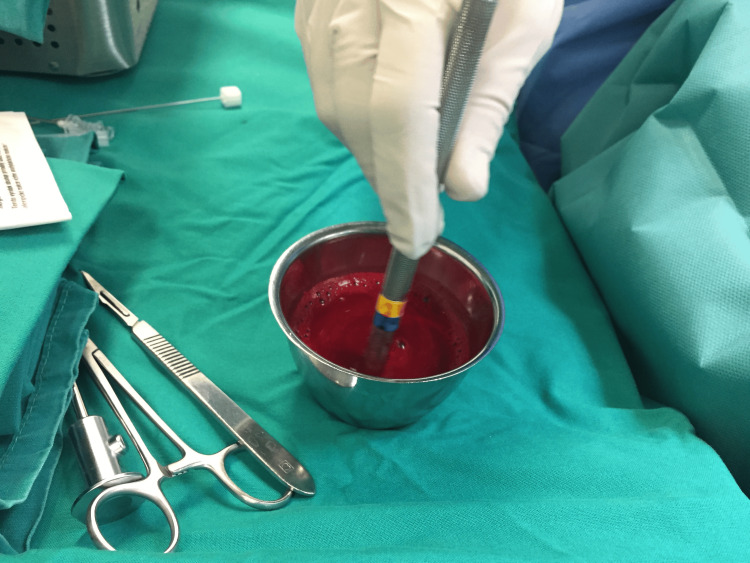
Fibrin clot formation procedure.

**Figure 6:**
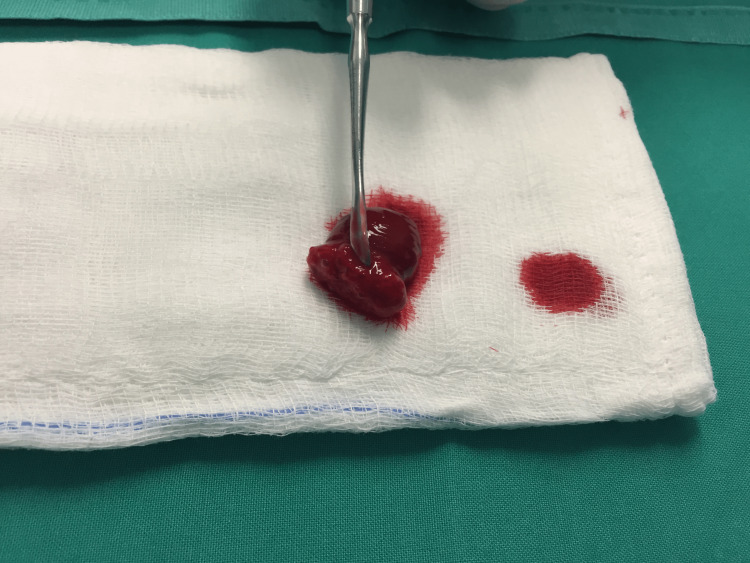
Placing formatted fibrin clot on to gauze.

In patients from the study group (Group B), meniscal repair was performed with the additional application of a fibrin clot. During the debridement of the meniscal lesion, the nursing staff conducted a blood draw from the subcutaneous veins of the upper limb, following meticulous antisepsis of the area. Subsequently, peripheral blood was collected in a sterile metal beaker and gently agitated with a blunt object, such as a glass syringe plunger, a 4.0 mm switching stick, or a metal cylinder, for approximately 15 minutes. This process resulted in the formation of a sufficient amount of fibrin clot at the peripheral end of the agitating object (Figures 5, 6) [5,6,11].

**Figure 7:**
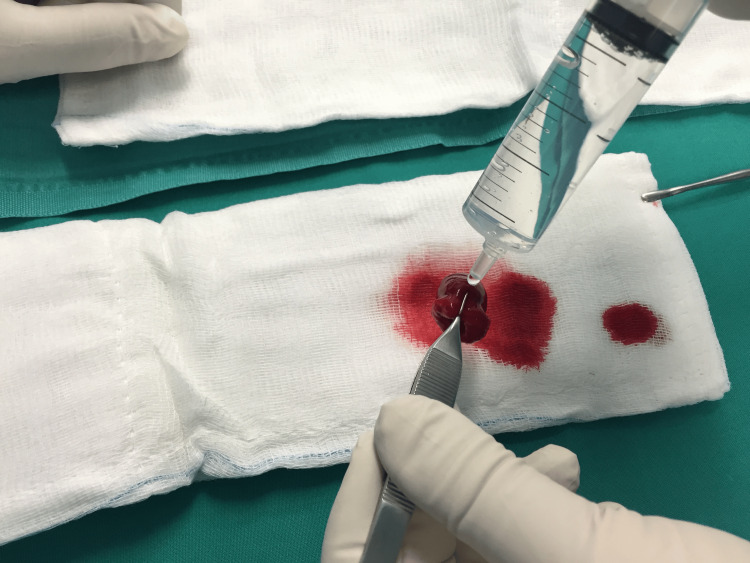
Rinsed fibrin clot with saline.

The formatted fibrin clot was delicately removed from the blunt object using Adson forceps and rinsed with saline (2-3 ml) to eliminate any peripheral blood that had not been incorporated into the fibrin clot (Figure 7).

**Figure 8:**
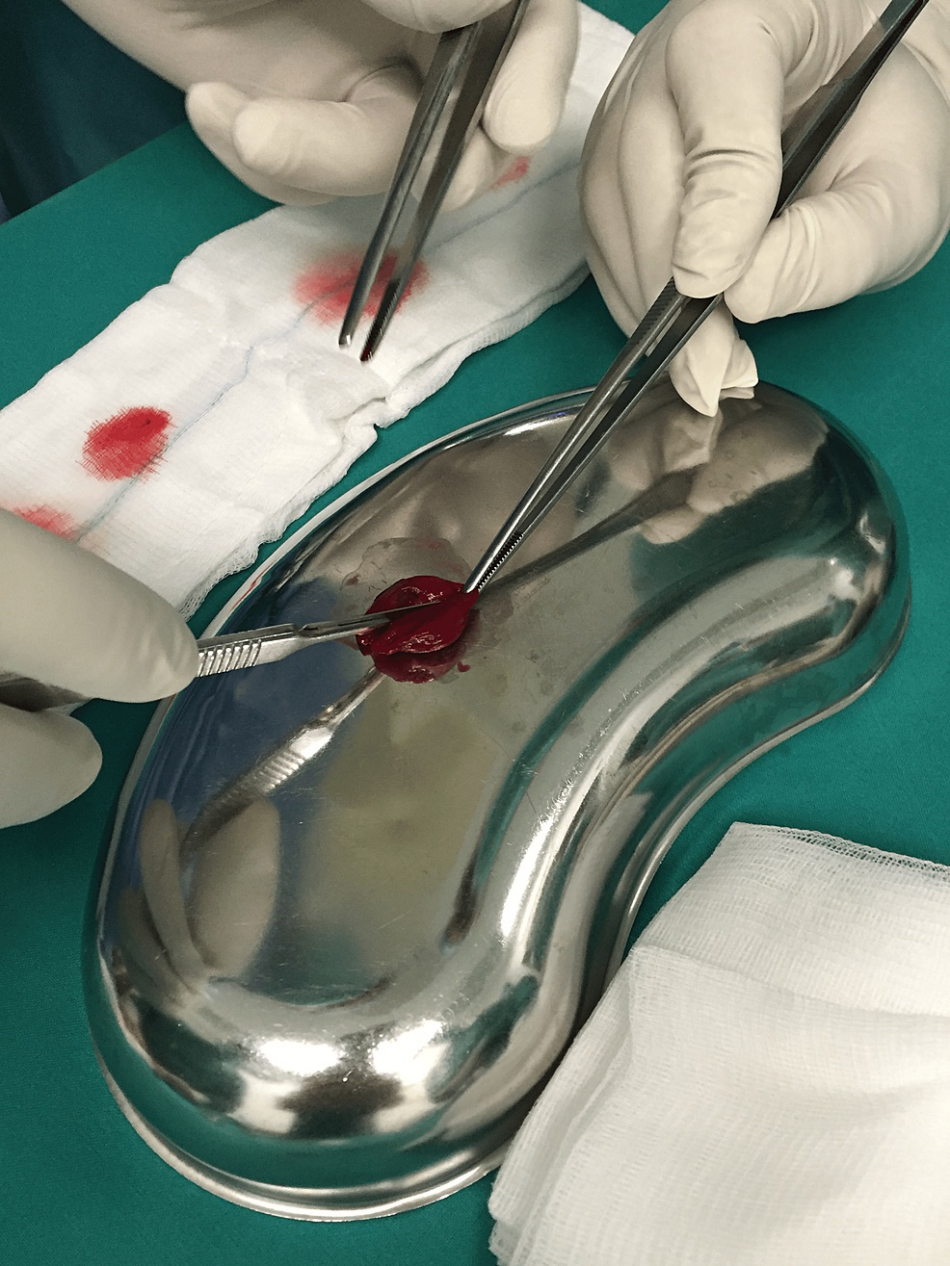
Shaping the fibrin clot.

**Figure 9:**
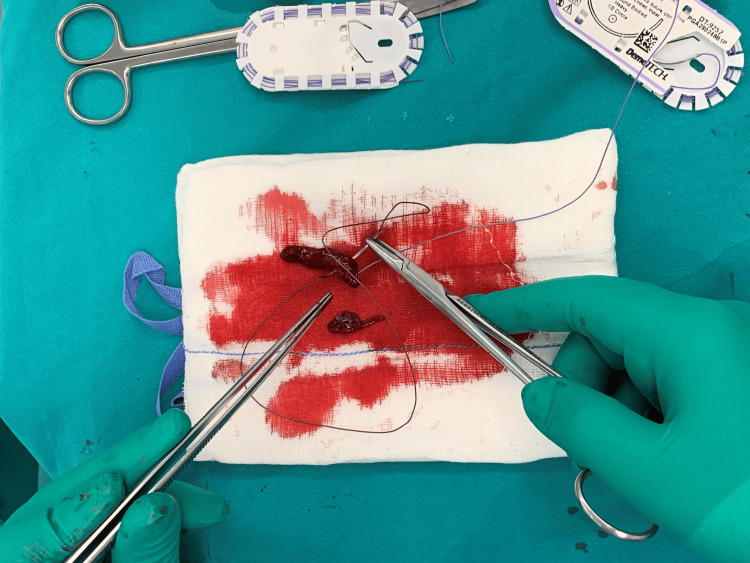
Placing the two absorbable sutures.

The removal of peripheral blood not only concentrated the fibrin clot but also had a significant impact on improving the arthroscopic view when the clot was placed intraarticularly. Subsequently, the clot was shaped with a blade (No. 11) according to the configuration of the meniscal lesion, and two absorbable sutures were placed at its distal ends to facilitate better control during its placement in the lesion (Figures 8, 9).

Following the placement of sutures, the next crucial step was to test the tensile strength and elasticity of the fibrin clot. Controlled forces were applied simultaneously to the two distal ends of the clot to evaluate its ability to withstand tension and stretching (Video 1). This crucial evaluation ensures that the fibrin clot is well prepared and capable of withstanding the mechanical stresses within the knee joint during the healing process.

**Video 1:** Testing the tensile strength and elasticity of the fibrin clot.

**Figure 10:**
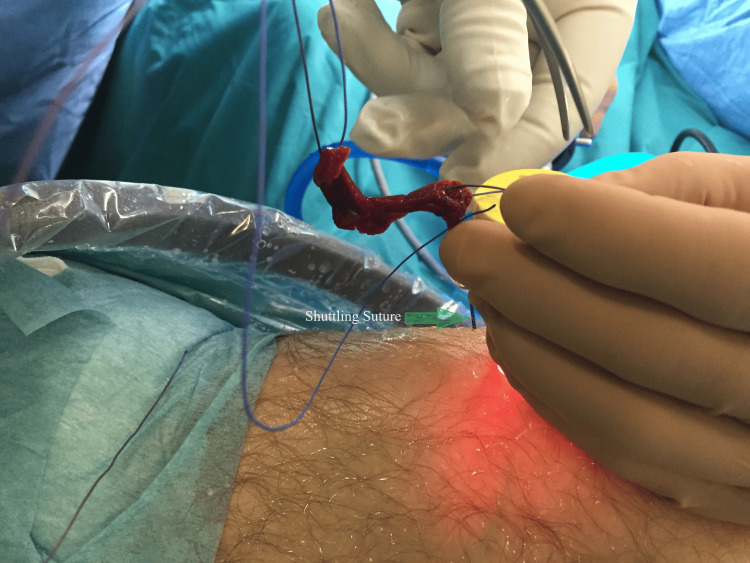
Management and handling of the fibrin clot in meniscal repair.

A critical phase in the surgical process involves the precise and gentle placement of the fibrin clot. To accomplish this, we secure one of the two distal ends of the vicryl sutures, integrated into the fibrin clot, to the end of the shuttling suture. Typically, the shuttling suture, usually a polydioxanone (PDS) suture, is introduced through an 18G needle from the arthroscopic portal, passing through the cannula to the location where the fibrin clot will be delivered intraarticularly. Gently pulling the shuttling suture ensures a controlled delivery of the fibrin clot directly into the meniscal gap. The fibrin clot is carefully shuttled through a cannula and guided through the arthroscopy portal. After completing the repair, the shuttling sutures are meticulously cut to conclude the procedure (Figure 10).

**Figure 11:**
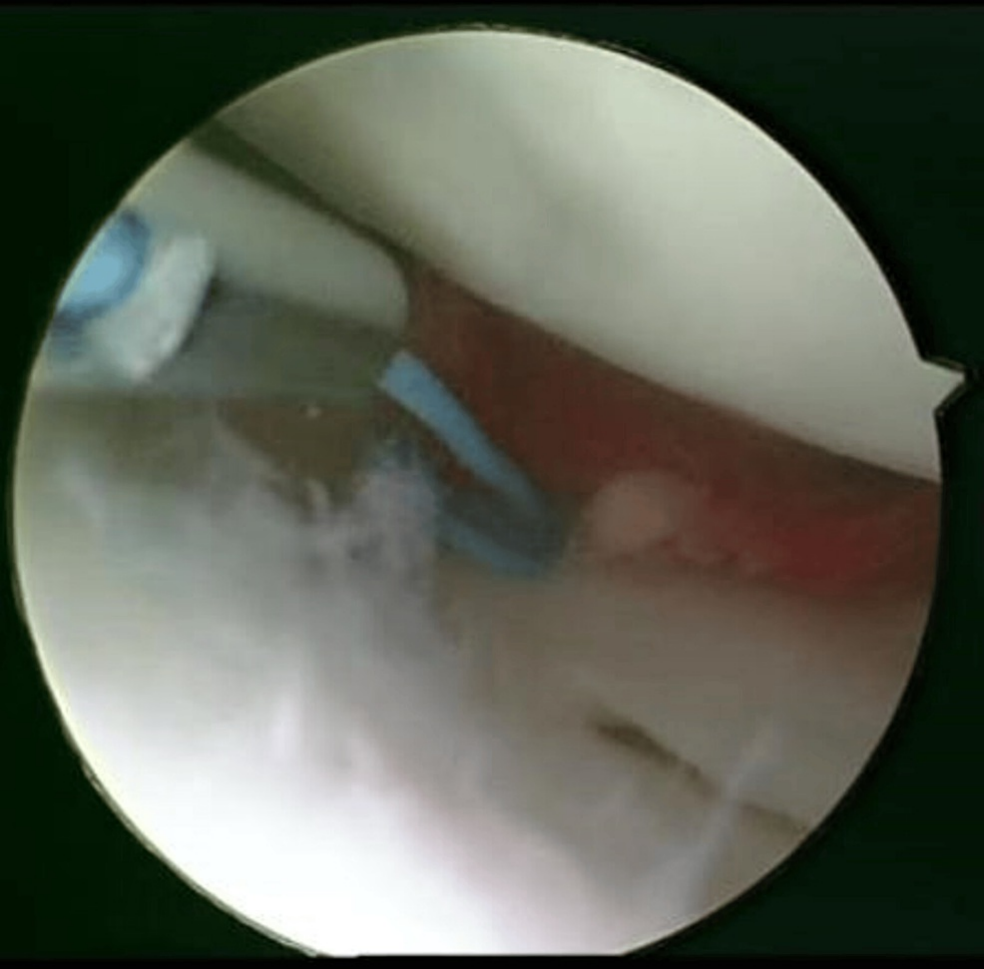
Fibrin clot was buried and stabilized.

The process of embedding the clot at the rupture site and precisely situating it within the area was skillfully executed using a probe. The closure of the meniscal gap and stabilization of the fibrin clot were meticulously achieved through suturing (Figure 11).

Post-surgical management guidelines

Following surgery, all participants, including those in Group A (the control group) and Group B (the study group), underwent an identical postoperative rehabilitation protocol, as outlined in the subsequent sections (Table 4). This standardized approach included the immediate placement of a functional brace to limit knee flexion to 0-60 degrees for two weeks. In the third week, flexion was progressively increased to 0-90 degrees, eventually reaching 0-120 degrees by the fourth week. Patients utilized axillary crutches for three weeks to prevent weight-bearing on the operated leg, followed by a transition to partial weight-bearing for one week and full weight-bearing clearance in the fourth week. Furthermore, patients initiated a physical therapy program on the first postoperative day, focusing on quadriceps femoris muscle strengthening exercises and a combination of passive and active exercises to enhance knee flexion, with modifications tailored to the specific postoperative week.

**Table 4:** Postoperative Rehabilitation Protocol The table outlines the Rehabilitation Protocol following the surgery, providing details on the duration and specific instructions for each phase. These phases guide patients through their postoperative recovery process, ensuring a safe and effective return to normal activities.

Rehabilitation phase	Time period	Instructions
Functional brace placement	Two weeks	Limit knee flexion from 0 to 60 degrees
Progressive increase in flexion	Third week	Increase flexion to 0-90 degrees
	Fourth week	Increase flexion to 0-120 degrees
Use of axillary crutches	Three weeks	Avoid loading the operated leg
Partial and full weight-bearing	After three weeks	Partial weight bearing for one week
	Fourth week	Full weight-bearing allowed
Physical therapy	From day 1	Strengthen quadriceps femoris muscle, passive and active exercises to improve knee flexion
Return to work	One to two weeks	Sedentary work allowed
	After three months	Allow returning to his normal working conditions as before with caution
Resumption of exercises	Six weeks	Low-intensity aerobic exercise allowed
	Three months	Jogging permitted
	Six months	Complete return to normal sports and activities permitted

The return to work and exercise was also carefully managed. Patients were allowed to return to sedentary work within one to two weeks post-surgery, while a cautious approach was taken for those wishing to resume their normal working conditions, with this typically occurring around three months after the procedure. This allowed patients to return to their regular work duties with a prudent approach to their pre-surgery working conditions. In terms of exercise, low-intensity aerobic activities were permitted after six weeks, with jogging becoming an option after three months. Finally, a complete return to normal sports and activities was allowed after six months.

The Rehabilitation Protocol we implemented is considered a gold standard for meniscal repair, as extensively described in the medical literature. This standardized approach ensures consistency in patient care and rehabilitation, contributing to the overall effectiveness of the treatment for both the control group (Group A) and the study group (Group B).

## Results

In the control group (Group A), the mean preoperative scores for the TLKSS, KSSS, and MCRSQ were 64±3, 55±4, and 33±3, respectively. These scores exhibited improvement at the end of the first postoperative three-month period (83±7, 80±9, and 75±8) and further enhancement after 12 months post-operation (93±7, 88±8, and 84±8, respectively) (Table 5).

**Table 5:** Preoperative and postoperative scores in the control group (Group A). TLKSS: Tegner Lysholm Knee Scoring Scale; KSSS: Knee Society Scoring System; MCRSQ: Modified Cincinnati Rating System Questionnaire.

Time point	TLKSS score (mean±SD)	KSSS score (mean±SD)	MCRSQ score (mean±SD)
Preoperative	64±3	55±4	33±3
Three months postoperative	83±7	80±9	75±8
Twelve months postoperative	93±7	88±8	84±8

The improvement was found to be statistically significant both before and after surgery at the three-month follow-up (p<0.001) and at the 12-month follow-up (p<0.001) in the study group (Group B) that received fibrin clots. The mean preoperative scores for the TLKSS, KSSS, and MCRSQ were 63±4, 57±4, and 31±4, respectively. These scores demonstrated improvement to 89±5, 82±7, and 83±7 after three months and further enhancement to 95±5, 91±8, and 89±8 after 12 months, respectively (Table 6).

**Table 6:** Preoperative and postoperative scores in the study group (Group B). TLKSS: Tegner Lysholm Knee Scoring Scale; KSSS: Knee Society Scoring System; MCRSQ: Modified Cincinnati Rating System Questionnaire.

Time point	TLKSS score (mean±SD)	KSSS score (mean±SD)	MCRSQ score (mean±SD)
Preoperative	63±4	57±4	31±4
Three months postoperative	89±5	82±7	83±7
Twelve months postoperative	95±5	91±8	89±8

The observed differences were statistically significant between 0 and three months of follow-up (p<0.001) and between three and 12 months of follow-up (p<0.001) (Table 7).

**Table 7:** Statistical significance of observed differences during follow-up periods. The p-value indicates the probability of observing the data, or something more extreme, under the assumption that the null hypothesis is true. p<0.001 indicates a probability of <0.1% that the observed differences are due to chance, suggesting very strong evidence against the null hypothesis.

Follow-up period	Statistical significance (p-value)
0-3 months	p<0.001
3-12 months	p<0.001

Comparison of the improvement in scores between the two groups, using an independent sample t-test, revealed significant differences in two out of the three questionnaires (TLKSS: p<0.001 and MCRSQ: p=0.004) (Table 8).

**Table 8:** Statistical significance of improvement in scores between groups. TLKSS: Tegner Lysholm Knee Scoring Scale; MCRSQ: Modified Cincinnati Rating System Questionnaire. The p-value indicates the probability of observing the data, or something more extreme, under the assumption that the null hypothesis is true. p<0.001: Very strong evidence against the null hypothesis, suggesting a highly significant difference. p=0.004: Strong evidence against the null hypothesis, indicating a significant difference.

Questionnaire	Statistical significance (p-value)
TLKSS	p<0.001
MCRSQ	p=0.004

The failure rate in the control group was 11.1% (three patients with a positive McMurray and Apley tests and poor scores three months postoperative). In the study group, the failure rate was 8.33% (two patients) (Table 9).

**Table 9:** Failure rates in control and study groups. Failure rate: The percentage of patients who did not respond positively to the treatment or intervention, as indicated by specific tests and assessments (e.g., positive McMurray and Apley tests and poor scores). Control group: The group of patients who did not receive the experimental treatment or intervention in the study. Study group: The group of patients who received the experimental treatment or intervention in the study.

Group	Failure rate	Number of patients
Control group (Group A)	11.1%	3
Study group (Group B)	8.33%	2

The examination and analysis of knee MRI conducted in the 12th postoperative month in the study group (Group B) yielded the following results: complete healing of the meniscal rupture in 22 of 24 cases (91.67%), partial healing in one case (4.17%), and failure of healing or resorption in one case (4.17%). In the control group (Group A), the findings were as follows: complete healing of the meniscal rupture in 19 of 27 cases (70.37%), partial healing in seven cases (25.93%), and failure of healing or resorption in one case (3.7%). Complete meniscal healing was significantly superior in the study group compared to the control group (p<0.05). Partial healing also exhibited significant improvement in the study group compared to the control group (p=0.033) (Table 10).

**Table 10:** MRI results of meniscal healing with statistical significance (12 months postoperative). MRI: magnetic resonance imaging. The p-value indicates the probability of observing the data, or something more extreme, under the assumption that the null hypothesis is true. p<0.05 indicates a statistically significant difference. p=0.033 indicates a statistically significant difference with moderate strength.

Outcome	Group B (study group, N=24)	Group A (control group, N=27)	Statistical significance
Complete healing	22 cases (91.67%)	19 cases (70.37%)	p<0.05
Partial healing	1 case (4.17%)	7 cases (25.93%)	p=0.033
Failure of healing/resorption	1 case (4.17%)	1 case (3.7%)	-

## Discussion

There are numerous surgical techniques available to facilitate and improve the healing process of meniscal ruptures [4]. In this study, we conducted a comparative analysis of the treatment approach involving simple stapling of meniscal ruptures using sutures versus the same method supplemented by the additional use of a fibrin clot. The rationale behind incorporating a fibrin clot in the meniscal rupture treatment is twofold [12,13]. Firstly, the fibrin clot offers chemotactic and mitogenic stimuli that promote tissue regeneration and reconstruction processes. Secondly, the fibrin clot serves as a scaffold upon which new tissue can develop. One potential drawback of applying a fibrin clot in the meniscal rupture area is the challenge of retaining it in the injured region without concurrent immobilization of the operative leg.

Our research offers several advantages compared to similar previous studies in this field. Notably, we included a relatively large sample size, comprising 24 patients. While this sample size is among the largest for this type of study, it is smaller than the study conducted by Jang et al. [14], which encompassed 41 meniscal tears, including 19 radial tears, 12 longitudinal tears in the red-white zone, seven transverse tears, and three oblique tears. However, the aforementioned study has certain weaknesses, such as (a) lack of reference to the age groups of the patients; (b) absence of a comparative assessment with a specific control group; (c) no clinical evaluation using clinical methods and/or specific clinical questionnaires; and (d) incomplete elucidation of the process of retesting and confirming meniscal healing (MRI or a follow-up arthroscopy). Additional studies related to our research include those conducted by Rodeo et al. [15], involving 17 meniscal ruptures, as well as studies by Henning et al. [16] and Ra et al. [17], both of which included 12 patients with meniscal ruptures where clot application was combined with simultaneous stitching of the meniscal rupture.

An innovative aspect of our research was the incorporation of a randomized control group with comparable age characteristics and morphological types of meniscal ruptures, ensuring a robust comparative assessment of the outcomes [18]. Unlike previous studies with only one-year follow-up evaluations postoperatively, our research included assessments at both the third and 12th postoperative months. This early three-month follow-up already highlighted the distinct advantage of the stitching technique with a fibrin clot over simple meniscus suturing, as evidenced by significantly improved TLKSS and MCRSQ clinical evaluation scores.

While the research showcased several strengths, such as a randomized control group and a detailed follow-up evaluation, there were acknowledged limitations, including a non-clustered age group analysis, a relatively short follow-up period, a lack of second-look arthroscopy for evaluating the meniscus healing process, and no assessment for different morphological types of rupture (Table 11).

**Table 11:** Limitations, impacts, and improvements in meniscal rupture treatment study. Limitation: This column identifies specific shortcomings or areas where the study on meniscal rupture treatment lacks depth or comprehensiveness. Impact: This column outlines the potential consequences or effects of each listed limitation on the study's results and their applicability. Improvement: This column suggests ways to enhance future studies, addressing the identified limitations to achieve more reliable and generalizable outcomes.

Limitation	Impact	Improvement
Non-grouping in age groups	May overlook nuances in different age groups, leading to non-universal conclusions.	Stratify participants into distinct age groups for detailed analysis
Short postoperative follow-up period (12 months)	Insufficient for observing long-term outcomes and complications	Extend the follow-up period to several years for comprehensive understanding
Lack of second-look arthroscopy for evaluating the meniscus healing process	Relies on less direct methods for assessing healing, potentially less accurate	Incorporate postoperative arthroscopy for a clearer picture of the treatment's effectiveness
No assessment for different morphological types of rupture	Study's conclusions may not apply equally to all types of meniscal injuries	Categorize and analyze outcomes based on the morphological characteristics of the rupture

Despite these limitations, the findings suggest that the use of fibrin clots in meniscal repair contributes to better clinical outcomes and enhanced healing rates, emphasizing the potential benefits of this augmentation technique in clinical practice.

## Conclusions

In conclusion, this study compared two methods for meniscal rupture repair, one involving simple suturing and the other incorporating the additional use of a fibrin clot. The research included a substantial sample size of 51 patients, with a follow-up evaluation at three and 12 months postoperatively. The results demonstrated significant improvements in clinical evaluation scores, including the TLKSS and MCRSQ, in the study group that received fibrin clots compared to the control group. Additionally, the failure rate was lower in the study group, indicating the efficacy of the fibrin clot augmentation.

Moreover, the MRI analysis at the 12th postoperative month revealed a higher rate of complete meniscal healing in the study group compared to the control group. The study addressed various morphological types of meniscal ruptures, including longitudinal and bucket-handle ruptures, providing valuable insights into the effectiveness of fibrin clots in different scenarios.

The findings support the potential benefits of incorporating fibrin clots into meniscal repair procedures and emphasize the importance of patient selection and meticulous surgical techniques.

In summary, the study provides compelling evidence for the benefits of fibrin clot augmentation in meniscal rupture repair, suggesting a significant step forward in treatment methods. Further research and long-term studies are warranted to refine and expand our understanding of the optimal use of an exogenous fibrin clot in meniscal repair, ultimately benefiting patients and advancing orthopedic surgery.
